# From Micro to Long: Non-Coding RNAs in Tamoxifen Resistance of Breast Cancer Cells

**DOI:** 10.3390/cancers13153688

**Published:** 2021-07-22

**Authors:** Jéssica Fernanda Barazetti, Tayana Shultz Jucoski, Tamyres Mingorance Carvalho, Rafaela Nasser Veiga, Ana Flávia Kohler, Jumanah Baig, Hend Al Bizri, Daniela Fiori Gradia, Sylvie Mader, Jaqueline Carvalho de Oliveira

**Affiliations:** 1 Post-Graduation Program in Genetics, Department of Genetics, Federal University of Parana, Curitiba 81530-000, Parana, Brazil; jessicabarazetti@ufpr.br (J.F.B.); tayanajucoski@ufpr.br (T.S.J.); tamyrescarvalho@ufpr.br (T.M.C.); rafaela.veiga@ufpr.br (R.N.V.); anakohler@ufpr.br (A.F.K.); danielagradia@ufpr.br (D.F.G.); 2 Department of Biochemistry and Molecular Medicine, University of Montreal, Montreal, QC H3T 1J4, Canada; jumanah.baig@umontreal.ca (J.B.); hend.al-bizri@umontreal.ca (H.A.B.); 3 Institute for Research in Immunology and Cancer, University of Montreal, Montreal, QC H3T 1J4, Canada

**Keywords:** breast cancer, estrogen receptor, tamoxifen, endocrine resistance, ncRNA, lncRNA, microRNA

## Abstract

**Simple Summary:**

Breast cancer is a disease that affects thousands of women around the world. Adequate treatment depends on the characterization of breast cancer subtypes. Tumors that are positive for the estrogen receptor represent the most common subtypes and have the best prognosis. However, many patients relapse due to resistance to tamoxifen, one of the main drugs used for these subtypes. In this study, our goal is to discuss a class of molecules that have been directly linked to the tamoxifen resistance process but is still underexplored: the non-coding RNAs (ncRNAs). We have reviewed ncRNAs that have been associated with different processes of resistance to tamoxifen in order to stimulate a discussion about the importance of knowing and understanding the role of these molecules in breast cancer and their relevance to clinical applications.

**Abstract:**

Breast cancer is the most commonly diagnosed cancer and the leading cause of cancer mortality among women. Two thirds of patients are classified as hormone receptor positive, based on expression of estrogen receptor alpha (ERα), the main driver of breast cancer cell proliferation, and/or progesterone receptor, which is regulated by ERα. Despite presenting the best prognosis, these tumors can recur when patients acquire resistance to treatment by aromatase inhibitors or antiestrogen such as tamoxifen (Tam). The mechanisms that are involved in Tam resistance are complex and involve multiple signaling pathways. Recently, roles for microRNAs and lncRNAs in controlling ER expression and/or tamoxifen action have been described, but the underlying mechanisms are still little explored. In this review, we will discuss the current state of knowledge on the roles of microRNAs and lncRNAs in the main mechanisms of tamoxifen resistance in hormone receptor positive breast cancer. In the future, this knowledge can be used to identify patients at a greater risk of relapse due to the expression patterns of ncRNAs that impact response to Tam, in order to guide their treatment more efficiently and possibly to design therapeutic strategies to bypass mechanisms of resistance.

## 1. Introduction

Breast cancer is the most commonly diagnosed cancer and the leading cause of cancer mortality among women [1]. Breast tumors can be classified in clinical practice as hormone receptor and/or HER2 positive (HR+ and/or HER2+) vs negative (HR- and/or HER2-) by immunohistochemical detection or as luminal A, luminal B, HER2-enriched and basal-like according to transcriptome profiling using gene signatures such as PAM50 [2]. Hormone receptors include the estrogen receptor alpha (ERα), the main driver of breast cancer cell proliferation, and the progesterone receptor (PR), a gene regulated by ER, both acting as hormone-dependent transcription factors. HR+ tumors are positive for ER and/or progesterone receptor (PR). The HR+HER2- subtype is the most common, representing around 73% of all occurrences, and generally has a good prognosis. The HR+HER2+ subtype has a higher cell proliferation index and usually a more aggressive phenotype, constituting 11% of occurrences [3].

Breast cancer classification and the development of targeted therapies has considerably improved treatment options and patient prognosis [4]. The subtypes that are positive for the estrogen receptor usually respond to endocrine therapies targeting the estrogen receptor (ER) pathway, based on antiestrogens, which include selective ER modulators (SERMs) and selective ER downregulators (SERDs), or on aromatase inhibitors (AIs), which prevent endogenous production of estrogens [5]. One of the most commonly prescribed first-line SERM for hormone responsive subtypes is tamoxifen [6]. Tamoxifen (Tam) is a highly efficient SERM widely used for treatment of all stages of breast cancer in pre- and post-menopausal women. It binds ER and blocks its transcriptional activity. Tam use in the clinic has led to a drop in the recurrence rate by 50% at 5 years and 30% lower during the next 5 years [7]. However, approximately 50% of advanced ER-positive breast tumors are intrinsically resistant to tamoxifen and about 40% of patients receiving adjuvant tamoxifen eventually relapse [8]. 

The mechanisms that are involved in Tam resistance are complex and involve multiple signaling pathways [9,10]. Recently, roles for microRNAs and lncRNAs in controlling ER expression and/or tamoxifen action have been described, but the underlying mechanisms are still little explored. In this review, we will discuss the current state of knowledge on the roles of microRNAs and lncRNAs in the main mechanisms of tamoxifen resistance.

## 2. Estrogens and Estrogen Receptor Alpha

Estrogens are mitogenic hormones that play crucial roles in normal breast development, but also in carcinogenesis. They are predominantly synthesized in the ovaries of premenopausal women and to a lesser extent in peripheral tissues, including breast tissue. In postmenopausal women, estrogens are only produced in extragonadal peripheral tissues [11].

The estrogen receptor alpha (ERα) is one of the most significant biological markers for the diagnosis/prognosis of breast cancer and its accurate detection is important for therapeutic choice in BC patients. ERα is member of the nuclear receptor superfamily of ligand activated transcription factors [12,13]. There are two functionally distinct ERs: ERα and ERβ. The human *ESR1* gene, which encodes ERα is located on chromosome 6 while the human *ESR2* gene, coding for ERβ, is located on chromosome 14 [14,15]. These receptors have different and often opposite effects and the proliferative response caused by estrogens in breast epithelial cells is thought to be the result of a balance between ERα and ERβ signaling. While ERα has a proliferative effect on breast cells, acting as a transcriptional activator of genes associated with cell survival and proliferation, the role of ERβ is usually antiproliferative and proapoptotic [16,17]. However, ERα is overexpressed in ER+ tumors while ERβ expression is reduced. 

The ERα is a 66 kDa nuclear protein and its transcriptional activity is ligand-dependent. Estrogens bind to the receptor and change its conformation, inducing binding to specific target DNA sequences called estrogen response elements (EREs) [12,13]. On DNA, the estrogen-ERα complex interacts with coregulatory proteins, modulating the transcription of genes involved in cell cycle regulation, DNA replication, cell differentiation, cell survival, and angiogenesis [14,18]. The estrogen-ER complex can also act on non-nuclear pathways through the regulation of membrane receptors (for example, IGFR, FGFR, and HER2) and kinases (for example, mitogen activated protein kinases, receptor tyrosine kinases, PI3K, AKT, mTOR, Src, and CDK4/6) [14,18].

The *ESR1* gene also encodes ER variants, such as ERα36 and ERα46 [19,20]. ERα36 has a novel noncoding exon as its first exon and also a unique 27 amino acid domain that replaces the last 138 amino acids in ERα66, and as a result lacks both transcription activation domains (AF-1 and AF-2) [21]. ERα46 is a truncated form that lacks the transactivation function domain 1 (AF1) and functions to inhibit the AF1 activity of the full length ERα66 [22]. 

## 3. Mechanisms of Tamoxifen Resistance

Despite the clinical efficacy of Tam, intrinsic or acquired resistance is an important obstacle limiting the success of ER + breast cancer patient treatment. It is a challenge that needs to be overcome to improve the prognosis of these patients. The main mechanisms of resistance to tamoxifen can be divided according to different causes: mechanisms that involve genetic mutations and lead to loss or gain of function of the receptor and mechanisms that modulate other protumorigenic pathways, including other receptors involved in estrogen’s pathway of action. Here we will highlight some of these mechanisms, with a focus on targets of non-coding RNAs implicated in resistance to tamoxifen.

A recently identified mechanism that interferes with the action of Tam is the emergence of mutations that alter the structure and function of the receptor. Constitutively active mutants can recruit coactivators in the absence of estrogens [23], leading to resistance to aromatase inhibitors, which act by depleting estrogen production. The efficacy of antiestrogens with these mutant ER forms is also reduced in a manner that is mutation and antiestrogen-specific [24]. On the other hand, since Tam’s function is mediated by binding to the ER, loss of ER expression and activation of ER-independent growth are also important mechanisms of tamoxifen resistance [25]. The latter can occur through amplification or overexpression of downstream effectors of the ER proliferative program, such as the transcription factor cMyc [26] or the cell cycle regulator CCND1 [27,28,29,30].

ER isoforms, without gene mutations, may also be associated with Tam resistance. Although tamoxifen is an antagonist of ERα66, it activates ERα36. This activation might play critical roles in intrinsic and acquired Tam resistance [31]. Additionally, the ER-α46 variant enhances sensitivity to estrogens in breast cancer cells [22]. 

Disruption of the balance between ERα coactivators and corepressors is associated with poor prognosis and Tam resistance. Tamoxifen induces a conformational change of ERα that blocks coactivator recruitment and favors the recruitment of corepressors. Some mutations, for example in corepressors, may be frequent in resistant cells [32].

Loss of ER may also be triggered by epigenetic mechanisms. For instance, ZEB1 and ZEB2, two transcription factors that trigger the epithelial mesenchymal transition (EMT), contribute to tumor malignancy [33]. Their suppression through in vitro regulation by miRNAs and lncRNAs was able to inhibit EMT and increase sensitivity of breast cancer cells to Tam [33]. HDACs (histone deacetylases), which remove acetyl groups of histones, can also modulate ERα expression [34]. Overexpression of *HDAC1* results in the loss of ERα in ER+ MCF7 breast cancer cells [35]. Intriguingly, treatment with HDAC inhibitors also suppresses ERα expression in ER+ breast cancer cell lines [36] while divergent conclusions have been reached concerning their impact on ER- cells [37,38].

**Figure 1:**
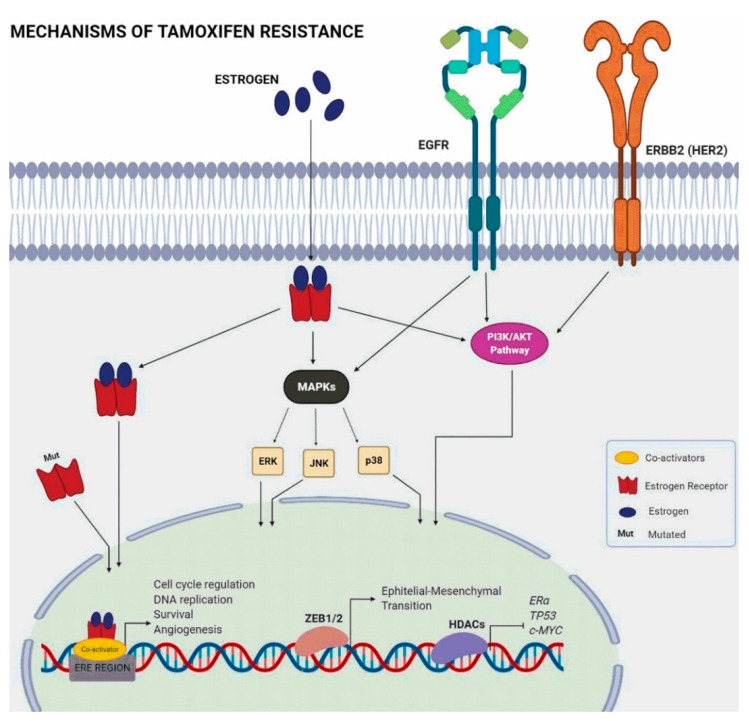
Estrogen signaling and pathways that interfere in tamoxifen resistance.

Some of the cellular mechanisms related to Tam resistance involve alternative oncogenic signaling pathways that can provide tumors with estrogen-independent stimuli for proliferation and survival (Figure 1). An example is the activation of the ERBB2 pathway (Erb-B2 tyrosine receptor kinase 2 or HER2), known to reduce sensitivity to tamoxifen [39,40]. RBP2 (retinoblastoma-binding protein 2), also known as KDM5A (lysine demethylase 5A), physically interacts with ER, increases the stability of the EGFR and HER2 proteins, and promotes activation of the PI3K/AKT pathway, inducing tamoxifen resistance [41].

The phosphoinositide 3-kinase/Akt/mammalian target of the rapamycin (PI3K/Akt/mTOR) pathway is a cell signaling pathway that plays an important role in controlling cell cycle, survival, and cell growth [42]. The PI3K/AKT/mTOR pathway is dysregulated in many types of cancers. In breast cancer, it can lead to the resistance to endocrine therapy [43]. Indeed, inhibiting this pathway improved the effectiveness of tamoxifen in cultured cells [44].

The MAPK (mitogen-activated protein kinase) pathways, including extracellular signal-regulated kinase (ERK), c-jun N-terminal kinase (JNK), and p38MAPK, also regulate many cellular functions including growth, differentiation, and survival as part of intracellular signaling cascades activated by membrane receptors. The suppression of ERK and p38 signaling increased cell sensitivity to tamoxifen in vitro [45].

Another resistance mechanism is associated with a lesser known estrogen receptor, the GPER (G-protein coupled estrogen receptor), which has a different structure from the two canonical ERs (ERα and ERβ). GPER is a transmembrane receptor capable of mediating proliferative signaling through binding of estrogen or antiestrogens and is expressed in 50–60% of breast cancer tissues [39]. Signaling through GPER activates metalloproteinases and induces the release of heparin-binding EGF, which binds and activates EGFR leading to downstream activation of signaling molecules, such as ERK1 and ERK2 [46]. 

Thus, defects in several signaling pathways can lead to resistance to Tam. The study of non-coding RNAs that interfere with either ER expression or with pathways involved in Tam resistance has added another layer of complexity to these mechanisms.

## 4. Non-Coding RNAs

Advances in sequencing technologies and computational approaches in the past few decades revealed that 75–85% of the genome is transcribed, although less than 3% of the human genome represents coding gene exons [47,48,49,50,51,52]. The remaining transcripts are non-coding RNAs (ncRNAs) and can be divided in subclasses. Ribosomal and transfer RNAs are the best known non-coding RNAs. However, in the past decade, increasing attention has been given to other classes of non-coding RNAs such as microRNAs, long non-coding RNAs, and circular RNAs, among others, notably for their roles in the regulation of gene expression and chromatin structure.

MicroRNAs (miRNAs/miRs) are small (approximately 18–25 nucleotide long) non-coding RNAs that, most of the time, interact with the 3′UTR of target mRNAs and post-transcriptionally regulate their expression by mRNA cleavage or by inhibition of translation [53]. Dysregulated miRNA expression is frequently associated with hallmarks in cancer development and resistance to therapies [54,55]. In breast cancer, several reports suggested that miRNAs might have an essential role in Tam resistance by the regulation of genes in previously described pathways [56,57,58,59,60]. 

Long non-coding RNAs (lncRNAs) are a class of non-coding transcripts that are over 200 nucleotide long [61,62]. LncRNAs have similarities with mRNAs in terms of length, transcription and splicing structure, yet lack protein-coding potential due to the absence of sizeable open reading frames, although some lncRNA may encode small functional peptides [61,63,64]. LncRNAs are located in intergenic, intronic or exonic loci, and can be imprinted. They may overlap with protein-coding genes in a sense or antisense direction [63,65,66,67,68].

There is growing evidence indicating that many lncRNAs are expressed in a temporal and tissue-specific manner and play a role in gene regulation through different mechanisms [62,66,67,68,69,70]. Accumulating evidence suggests that some lncRNAs may be key regulators of biological processes like imprinting [71,72], pluripotency [73,74,75,76], cell differentiation [74,75], DNA damage response [68], cell apoptosis [70], inflammatory and immune responses [77]. Mutations, polymorphisms or altered expression patterns of lncRNAs have been associated with the progression and/or severity of several diseases, including breast cancer [68,78,79,80,81]. More recently, lncRNA deregulation has also been associated with Tam resistance in breast cancer cells [82].

## 5. Integrative Review: Methodology

**Figure 2:**
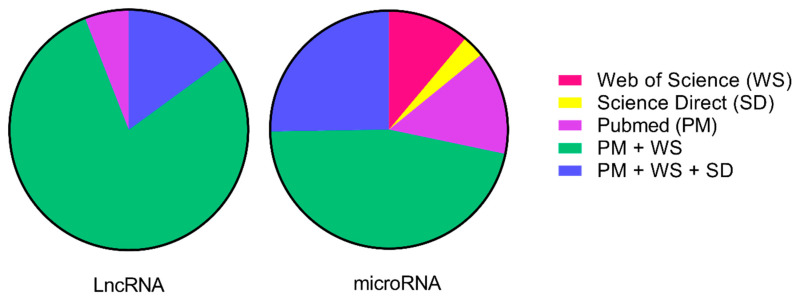
Percentage of articles found in one or more literature sources. A literature search was performed on PubMed (PM), Science Direct (SC), Medline (MD), and Web of Science (WS) databases using the following keywords: “long non-coding RNA/lncRNA” OR “microRNA/miRNA/miR” AND “tamoxifen resistance/tam-resistance/endocrine resistance”. We represented the final numbers of publications selected in this review. The Medline source had no studies after applying the exclusion criteria.

An integrative review of the literature on miRNAs and lncRNAs involved in tamoxifen resistance was performed. A literature research on PubMed, Science Direct, Medline and Web of Science database using the following keywords: “long non-coding RNA/ lncRNA” OR “microRNA/miRNA/miR” AND “tamoxifen resistance/tam-resistance/endocrine resistance” was done by more than one author. The inclusion criterion was original research papers with lncRNA or miRNA related to tamoxifen resistance. Case reports, review articles, and meta-analyses were not considered and articles repeated in several databases were counted only once (Figure 2). Thereby, we obtained lists of miRNAs and lncRNAs possibly related to tamoxifen resistance. Both lists were then evaluated according to title, abstract, and full text. The articles wherein miRNAs/lncRNAs were not the focus or where these molecules were not properly evaluated or not evaluated in the context of tamoxifen resistance were excluded. 

**Figure 3:**
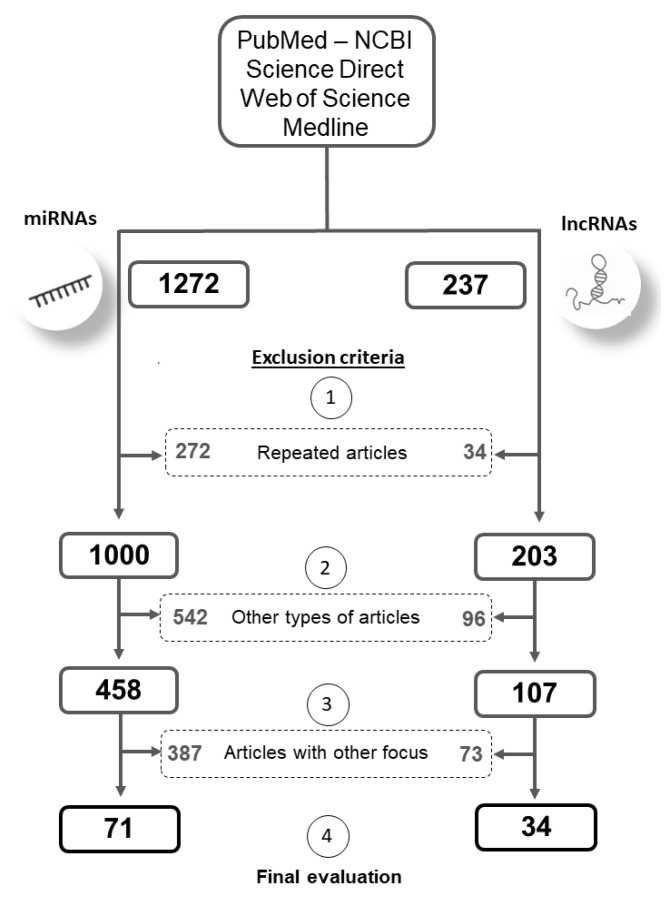
Schematic representation of study selection divided by microRNAs and long non-coding RNAs in four databases (PubMed, ScienceDirect, Medline, and Web of Science). Keyword variants were used to perform the search (long non-coding RNA; lncRNA; microRNA; miRNA; miR) AND (tamoxifen resistance; tam-resistance; endocrine resistance). Selection was performed based on inclusion criteria: original research papers with miRNA or lncRNA related to tamoxifen resistance, and exclusion criteria: (**1**): repeated articles between databases that were counted more than once; (**2**): articles not classified as original research papers, such as: case reports, reviews, book chapters, and meta-analyses; (**3**): papers wherein miRNAs/lncRNAs were not the focus and these molecules were not properly evaluated. The values inside the central dotted rectangles are the numbers of articles excluded in each selection step, and the external rectangles comprise the final numbers after each selection step. In the end, we obtained two lists of articles for miRNAs and lncRNAs that were analyzed individually (**4**).

All steps and exclusion criteria and the numbers of publications selected at each step for analysis in this review are listed in Figure 3.

## 6. Results

After a systematic review of the literature according to our research strategy, we gathered information about 75 different miRNAs in a total of 71 studies and about 22 different lncRNAs in a total of 34 studies. Detailed information about these ncRNAs is summarized in Table 1 (miRNAs) and Table 2 (lncRNAs).

### 6.1. microRNAs and Tamoxifen Resistance

Mechanistic studies about Tam resistance have been conducted mainly in breast cancer cell lines, in which resistance can be induced by long term culture in the presence of Tam or its active derivate 4-hydroxy-tamoxifen. Several studies [83,84] mapped differentially expressed miRNAs in matched resistant and sensitive ER+ breast cancer cells, such as MCF-7/MCF-7R. Most MCF7-R cells were generated with long-term exposure to 4-hydroxytamoxifen [85]. 

Almost a quarter of the selected studies involving miRNAs highlighted the miR-221/222 family, the first and most studied oncomiR cluster related to tamoxifen resistance. Tamoxifen resistant cells exhibit increased expression of miR-221/222 and knockdown of miR-221/222 restores cell sensitivity [86,87,88]. MiR-221 and miR-222 interact with the 3′-UTR of the *ESR1* gene and decrease ERα mRNA and protein levels [87,89,90,91,92,93,94]. Interestingly, miR-221/222 expression is conversely downregulated by ERα agonists, suggesting the existence of a reciprocal negative feedback [92]. However, in addition to *ESR1*, miR-221/222 also downregulates other genes such as *p27Kip1* and *PTEN,* which may explain their proliferative effects in Tam resistant cells. ERBB2 signaling further led to increased levels of miR-221/222 and decreased levels of ERα [86,92]. 

*ESR1* is also a known target of several miRs besides miR-221/222 [87,89,90,94]. Altered expression of these molecules could be an advantage for tumor progression, especially in combination with the activation of oncogenic pathways. Kim and colleagues found that upregulation of miR-500a-3p promotes tamoxifen resistance by downregulating *ESR1* [89]. Other miRNAs overexpressed in tamoxifen-resistant cells and having *ESR1* as a direct target include miR-335-3p and miR-335-5p [90]. 

On the other hand, miR-135a, which directly targets *ESR1*, was downregulated in Tam resistant cells [94]. Downregulation of miR-192-5p promotes tamoxifen resistance by upregulating *LY6K* [89] and miR-873 is downregulated in tamoxifen-resistant cells, targeting CDK3, which phosphorylates Serine sites of ERα [57]. The induced expression of miR-873 inhibited the ERα transcriptional activity in MCF7/Tam R cells and sensitized these cells to tamoxifen. Additionally, the injection of cells expressing miR-873 into nude mice treated with tamoxifen inhibited the tumor growth by increasing the sensitivity to tamoxifen in vivo [57]. 

Some microRNAs may target ER isoforms and modulate Tam resistance. ER-α36, upregulated in resistant cells, is a target of let-7 and induced let-7 expression in Tam-resistant MCF7 cells, downregulated ER-α36 expression and enhanced the sensitivity of MCF7 cells [95]. Additionally, the CUL4B protein influences Tam-resistance of breast cancer cells by upregulating ER-α36 expression, which was mediated by downregulation of miR-32-5p [96]. A larger number of miRNAs have been found to regulate *ESR1* expression but without reported roles in tamoxifen response have not been included in this review. 

Another important protein regulated by miRs acting as mediator of tamoxifen resistance is ERBB2/ HER2 [97,98,99]. Reduced expression of miR-26a/b, which can regulate *ERBB2* levels post-transcriptionally through interaction with its 3′UTR, correlates with *ERBB2* upregulation in MCF7/TAMR and T47D/TAMR cells, derived by long-term exposure to tamoxifen [40]. 

PTEN (phosphatase and tensin homolog) acts as a tumor suppressor and is an important negative regulator of the PI3K signaling pathway. Alterations in PTEN occur frequently in breast cancer cells and promote cell growth, survival, and migration [100]. Some miRs such as miR-29a/b, miR-146b, miR-301, and miR-519a have *PTEN* as a target and thus might influence tamoxifen therapy outcomes [101,102,103,104]. MCF-7 tamoxifen-resistant cells can express high levels of miRNA-519a, which directly represses the expression of *PTEN* and induces cell proliferation [102]. Higher levels of miR-221/222 also downregulate *PTEN*. On the other hand, some miRs have reduced levels in Tam resistant cells. Phuong and colleagues [103] studied miR-146a/b expression levels in tamoxifen-resistant MCF7 derived by long-term exposure. They observed that miR-146b can restore *PTEN* expression by suppressing *PTEN* promoter methylation in resistant cells. Furthermore, miR-146b overexpression inhibited cell proliferation and restored tamoxifen-sensibility in MCF7/TAMR [103].

Finally, some authors found that miRs could target histone deacetylases (HDACs). Although the role of these molecules in the induction of hormone therapy resistance in breast cancer cells remains uncertain, HDACs dysregulation stimulates cancer cell survival [105]. Studies demonstrated that miR-10b and miR-125a are associated with *HDAC4/HDAC2* and *HDAC5* expression, respectively [56,105]. These miRNAs seem to influence invasion, migration, and metastasis, in addition to drug resistance of breast cancer cells. Ahmad and colleagues [56] observed that overexpression of miR-10b, in ER+ cells increases resistance to tamoxifen and there is a negative correlation between *HDAC4* and miR-10b expression. Downregulation of *HDAC4* is induced by miR-10b, and the authors propose miR-10b-HDAC4 as a molecular mechanism related to tamoxifen resistance. 

Huang and colleagues also reported that resistant MCF7-TamC3 (created by prolonged culture under estrogen-deprived conditions) demonstrated decreased expression of miR-125a-5p, but increased expression of *HDAC2* and *HDAC5* compared to the parental tamoxifen-sensitive cells [105]. These outcomes suggest that dysregulations of *HDAC2*, *HDAC4*, and *HDAC5* can collaborate with the development of tamoxifen resistance in ER+ breast cancer cells associated with miR-10b and miR-125a-5p deregulation. 

Downregulation of the tumor suppressor miR-125a-5p was correlated with poor relapse-free survival (RFS) in tamoxifen-treated BC patients [105]. Induced expression of miR-125a-5p and also of miR-27b-3p, miR-29b-1, miR-135a, miR-500a-3p, miR-186-3p, miR-320a, and miR-4653-3p in breast cancer cell lines restored the sensitivity to tamoxifen, suggesting that these miRs are important as potential therapeutic options [89,105,106,107,108,109,110]. 

Using in vivo models, He and colleagues reported that restoration of miR-186-3p by systemic delivery of cholesterol-modified agomiR-186-3p to mice with T47D-tamoxifen R cells implantation suppressed tumor growth. Its role in the tamoxifen response and cell metabolism in ER+ breast cancer cells is promoted by the miR-186-3p/EREG/EGFR axis [110]. Likewise, the induced expression of miR-320a in MCF7-Tam R cells implanted in nude mice treated with tamoxifen inhibited tumor growth by restoring tamoxifen sensitivity [106]. The transference of exosomal miRNAs to cancer cells has also been studied. Liu and colleagues showed that injection of exosomes derived from MCF-7/TAM resistant cells transfected with miR-9-5p inhibitor into nude mice reduced the tumor volume and increased cell apoptosis [111,112].

Several other microRNAs with different target genes were also reported to play a role in tamoxifen resistance, as shown in Table 1.

**Table 1:** microRNAs associated with tamoxifen-resistance.

microRNA	Targets	Reference
Upregulated		
miR-9-5p	*ADIPOQ, ESR1, NCOA3*	[111,112]
miR-10b	*HDAC4*	[56]
miR-15b	*FOXO1*	[113]
miR-18a	*ESR1*	[114]
miR-21	*PDCD4, TPM1*	[86,115,116,117]
miR-22	*ESR1, PTEN*	[118]
miR-23b-3p	*SLC6A14, ZBTB1*	[119,120]
miR-24-3p	*BIM*	[84]
miR-92a-3p	*C21orf91, GPM6A, KAT2B, NEDD4L, UBE2Z, ROBO2, PCDH11X, PDS5B, TMEM87A, PRKCE*	[121]
miR-101	*MAGI-2*	[122]
miR-140	--	[122]
miR-155	*SOCS6*	[123]
miR-181	--	[86]
miR-192-5p	ESR1	[89]
miR-221/222	*P27*, *ESR1*, *TRPS1*	[86,87,88,93]
miR-301	*PTEN, FOXF2, COL2A1, BBC3*	[101]
miR-335-3p; miR-335-5p	*ESR1*	[90]
miR-342	--	[124]
miR-342-3p; miR-342-5p	*NR4A2, MAGED2, LASP1, UCP2, THSD4, PRODH, PIP4KSC, MEIS1, BMP7, PRR6, GEMIN4, GJAI, SEMA3D*	[117,124]
miR-335-5p, miR-335-3p	*ESR1*	[90]
miR-380	--	[122]
miR-489	--	[86]
miR-497	--	[125]
miR-519a	*CDKN1A, RB1, PTEN*	[102,125]
miR-542-5p	*YWHAB, LY9, SFRP1*	[126]
miR-552	--	[122]
miR-575	*CDKN1B*	[127]
miR-578	--	[122]
miR-599	--	[122]
miR-663b	*TP73*	[128]
miR-671-3p	*HNRNPA2B1*	[129]
miR-1266-5p	*HNRNPA2B1*	[129]
miR-1268a	*HNRNPA2B1*	[129]
miR-1280	--	[125]
Downregulated		
let-7	*ESR1* (ER-α36 isoform)	[130]
miR-15a	*BCL2, CCNE1*	[124,131]
miR-16	*BCL2, CCNE1*	[124,131]
miR-21	*PTEN, PDCD4*	[132]
miR-26a	*E2F7, EZH2, ERBB22H1*	[40]
miR-27a;miR-27b-3p	*HMGB3, NR5A2, CREB1*	[59,107,133]
miR-29a;miR-29b-1	*DICER1, PTEN*	[104]
miR-29a-3p; miR-29b-3p	*HNRNPA2B1, ATP5G1, ATPIF1*	[104,129]
miR-32-5p	*CUL4B*	[96]
miR-101	--	[125]
miR-106a	*CDKN1A, PTEN, RB1*	[125]
miR-125a	*CYP4Z1, CYP4Z2P, CDK3, HER2, HER3*	[108,134]
miR-125a-3p; miR-125a-5p	*HDAC5*	[105,109]
miR-125b-5p	*PAD2*	[135]
miR-135a	*ESR1, ESRRA, NCOA1*	[94]
miR-146a; miR-146b	*NFKB1, PI3K, TRAF6, IRAK1, PTEN*	[103,117]
miR-148a	*ALCAM*	[60]
miR-152	*ALCAM*	[60]
miR-181	--	[125]
miR-186-3p	*EREG*	[110]
miR-193a/b-3p	*ESR1, NCOA3*	[112]
miR-196a	*HOXB7*	[136]
miR-200	*ZEB1/2*	[137]
miR-214	*UCP2*	[58]
miR-222-3p	*ESR1, CDKN1B, HNRNPA2B1*	[129]
miR-320a	*ARPP19, ESRRG*	[106]
miR-342	*TXNIP, SEMAD, BMP7, GEMIN4*	[138]
miR-363	*SERTAD3*	[139]
miR-375	*HOXB3, MTDH*	[102,140]
miR-378a-3p	*GOLT1A*	[141]
miR-449a	*ADAM22*	[142]
miR-451	14-3-3ζ	[143,144]
miR-484	*KLF4*	[145]
miR-500a-3p	*LY6K*	[89]
miR-574-3p	*CLTC*	[134]
miR-873	*CDK3*	[57,146]
miR-877	--	[134]
miR-4653-3p	*FRS2*	[108]

Note: Regulation (up or down) of miRNAs in tamoxifen resistant breast cancer cells is indicated as reported. (--) information not provided in the study.

### 6.2. Long Non-Coding RNAs and Tamoxifen Resistance

Growing evidence suggests a role for some lncRNAs in endocrine therapy resistance for estrogen receptor-positive breast cancer. Regarding tamoxifen specifically, most of the studied lncRNAs that have been associated with the resistance process are upregulated. The expression of lncRNA LINC-ROR (ROR, regulator of reprogramming), for example, is positively associated with tamoxifen resistance [42,147,148]. LINC-ROR knockdown through in vitro and in vivo experiments indicated that this lncRNA may also contribute to cancer progression, since its inhibition reduces cell proliferation, invasion and migration [148], while LINC-ROR knockout enhanced breast cancer cell sensitivity to tamoxifen [147]. 

The mechanisms by which LINC-ROR might promote Tam resistance are not fully elucidated, but they might be related to inhibition of autophagy, since upregulation of LINC-ROR is negatively associated with *MAP1LC3A* (coding for LC3) and *BECN1* (coding for Beclin I) expression [148]. LINC-ROR may also be involved in estrogen receptor activation through the MAPK/ERK pathway, specifically by facilitating the degradation of the ERK-phosphatase DUSP7 [147]. In addition, LINC-ROR knockdown and overexpression experiments demonstrated that it might have an important role in EMT by acting as a sponge to miR-205-5p and, in consequence, positively regulating the ZEB1 and ZEB2 transcription factors [33]. Combined siLINC-ROR and tamoxifen treatment inhibited the tumor growth and metastasis of BT474 cells in nude mice [148].

H19 is one of the most studied lncRNAs in breast cancer, and its upregulation in tamoxifen resistance has been explored in multiple studies [85,149,150]. In resistant cells, H19 silencing increased the sensitivity to tamoxifen [85,149,150], observed by a decrease in cell proliferation [150], invasion ability [150], cell survival [149,150] and an increased in apoptosis [150]. In addition, H19 downregulation inhibited the Wnt/β-catenin pathway and EMT process [150]. Other processes are highlighted in H19-mediated resistance response. Unlike what has been described for ROR, the H19 lncRNA exhibits a positive correlation with Beclin-1 and LC3-II expression, and a strong induction of the autophagy process in resistant cells [85]. Autophagy exhibits dual roles in cancer development. This mechanism is involved in cell protection to clear damaged organelles and remove products of metabolism. However, this process also takes part in cancer cell chemotherapy resistance as a protective mechanism to survival [151,152,153]. H19 appears to regulate autophagy through the H19-SAHH-DNMT3B axis that promotes Beclin-1 promoter methylation [85]. In another mechanism, Tam resistance is reversed by the downregulation of H19 through the inhibition of the Notch and c-Met signaling pathway. The development of resistance to tamoxifen in endocrine therapy-resistant cells can also be related to H19-mediated regulation of ERα expression [149].

Another highlighted lncRNA is the urothelial carcinoma-associated 1 (UCA1) transcript, which may also enhance tamoxifen resistance [154,155,156,157,158] and is correlated with breast cancers outcome [155]. Interestingly, Tam treatment itself, through *HIF1A* upregulation, might enhance UCA1 expression, which in turn seems to increase tamoxifen resistance [154]. There might be a regulatory feedback loop concerning UCA1, since it may act as a sponge for miR-18a, which is a negative regulator of *HIF1A* [154]. Furthermore, UCA1 is positively associated with cell viability [154,157], cell survival cell colony formation [154], migration [155], and cell cycle progression [154,159], while it is negatively related to cell apoptosis [154,155,156,157]. LncRNA UCA1 may activate the PI3K/AKT pathway by inducing cell cycle progression through the phosphorylation of CREB through AKT [159] and preventing apoptosis through the activation of mTOR [156]. In addition, another suggested mechanism by which lncRNA UCA1 may promote tamoxifen resistance involves the nuclear translocation of β-catenin in the Wnt/β-catenin pathway [155].

Upregulation of CCAT2 (colon cancer-associated transcript 2) lncRNA was observed in breast cancer tissues and cell lines, besides being associated with poor prognosis [160,161]. In addition, tamoxifen-resistant cells also exhibited higher CCAT2 expression [162]. This lncRNA may be related to tamoxifen resistance by hyper-activating the ERK / MAPK pathway. CCAT2 knockdown experiments showed decreased cell proliferation, increased apoptosis rates, and improved sensitivity to tamoxifen [162]. 

DSCAM-AS1 (down syndrome cell adhesion molecule-antisense RNA 1) is a lncRNA whose gene contains estrogen response elements [163]. Its expression is induced in the response to estrogens and reversed with tamoxifen treatment [155]. DSCAM-AS1 is upregulated in breast cancer tissues and in tamoxifen resistant cells and positively associated with high grade and metastasis [164]. Additionally, silencing experiments decreased cell proliferation [164,165], increased apoptosis rate, and induced cell cycle arrest at G0 / G1 [165]. The RNA binding-protein hnRNPL interacts with DSCAM-AS1 3’-end and its knockdown also reduced cell proliferation in resistant cells. Thus, hnRNPL participates with DSCAM-AS1 in tamoxifen-resistance response [164]. There is no consensus regarding how DSCAM-AS1 promotes resistance in ER-positive breast cancers under tamoxifen treatment. One of the mechanisms involves regulating the epidermal growth factor receptor pathway substrate 8 (*EPS8*) gene [165], since *EPS8* silencing has the same effects as suppressing DSCAM-AS1. In this regulatory way, DSCAM-AS1 sponges miR-137, precluding *EPS8* regulation [165]. 

Overexpression of HOTAIR has been observed in Tam resistant breast tumors when compared to primary ones, and its downregulation significantly suppressed growth in Tam resistant model cell lines, suggesting it may represent a therapeutic target for Tam-resistant tumors. HOTAIR is directly repressed at the transcriptional level by estrogen-activated ER and is overexpressed in estrogen-depleted or tamoxifen-treated cells. HOTAIR can interact with ERα to increase its genomic signaling by increasing ERα protein levels. The effects were more prominent in the absence of ligand, suggesting a role of HOTAIR in regulating ligand-independent ER signaling [166,167]. In addition, HOTAIR has also been reported to interact with the histone modifying complexes polycomb repressive complex 2 (PRC2) and LSD1/COREST/REST, which may silence tumor suppressor genes such as PTEN, making HOTAIR a potential broad therapeutic target [168,169,170]. Another lncRNA negatively regulated by ER signaling is LINP1, whose knockdown increased the tamoxifen sensitivity in vivo [171].

Recently, the lncRNAs BDNF-AS [172], MAFG-AS1 [173] and DILA 1 [174] were also associated with Tam resistance. BDNF-AS overexpression promotes Tam resistance through the BDNF-AS/RNH1/TRIM21/mTOR pathway [172]. The MAFG-AS1 is upregulated in BC samples [173,175,176,177] and luminal subtype patients [173]. The MAFG-AS1 knockdown in luminal cell lines, MCF-7 and T47D, decreased cell proliferation, colony formation and promoted cell cycle arrest at G1. MAFG-AS1 is related to cell proliferation due to its function as competing endogenous RNA (ceRNA) of miR-339-5p which regulates CDK2 expression [173]. The knockdown of MAFG-AS1 and CDK2 decreased the proliferative capacity of MCF7 resistant-cells cultivated with tamoxifen. Thus, MAFG-AS1 and CDK2 promoted tamoxifen resistance. The knockdown of the *ESR1* gene in T47D cells upon E2 treatment revealed that MAFG-AS1 expression is regulated by ERα, within binds to the MAFG-AS1 promoter. The oncogenic role of MAFG-AS1 in the luminal subtype is dependent of the ERα transcription factor [173].

The novel lncRNA DILA1 (cyclin-D1 interacting long noncoding RNA 1) was identified as a regulator of cyclin D1 levels in MCF7 and T47D tamoxifen-resistant cells [174]. DILA1 knockdown decreased cell growth, arrested the cell cycle at G1/S and reversed tamoxifen resistance. In addition, DILA1 higher expression was associated with the clinical stage and with lymph node metastasis [174].

On the other hand, some lncRNAs such as ADAMTS9-AS2 and GAS5 seem to promote Tam resistance only when downregulated. Both of them act as sponges of miR-130a-5p and miR-222, respectively [178,179]. These miRNAs are negative regulators of *PTEN* expression. PTEN acts by blocking the PI3K/AKT signaling pathway and thus inhibiting AKT-mediated oncogenic signaling [180]. Downregulation of *PTEN* was previously related to tamoxifen-resistance in the MCF-7 breast cancer cell line overexpressing the tumor suppressor gene *MTDH* [181]. 

ADAMTS9-AS2 (ADAMTS9 antisense RNA 2) was described in chemoresistance in some types of cancer, including renal and glioblastoma [182,183], besides breast cancer. In Tam-resistant breast cancer tissues and cell lines, ADAMTS9-AS2 is downregulated [178]. ADAMTS9-AS2 overexpression decreased the viability of MCF7-R cells and increased apoptosis. The expression levels of this lncRNA and of the *PTEN* tumor suppressor gene are positively correlated in breast cancer cells. Furthermore, ADAMTS9-AS2 may act as a sponge of miRNA-130a-5p in this regulatory network [178]. 

GAS5 (growth arrest-specific transcript 5) is another lncRNA that exhibits a positive correlation with PTEN expression in tamoxifen-resistant cells [179]. The low expression of GAS5 is related to the increase in cell proliferation and the invasion ability and a decrease in apoptosis. On the other hand, GAS5 upregulation allows tamoxifen sensitivity by acting as a sponge for miR-222 [179]. In vivo experiments with MCF7-R cells treated with tamoxifen and transfected with a vector overexpressing a *GAS5* cDNA into nude mice decreased tumor growth [179]. This effect highlights the potential of GAS5 upregulation in restoring tamoxifen sensitivity.

Other lncRNAs described in tamoxifen resistance are summarized in Table 2.

**Table 2:** LncRNAs associated with tamoxifen resistance.

lncRNA	Ensembl Gene ID	Mechanism/Pathway	Reference
Upregulated			
ATXN8OS	ENSG00000230223	miR-16-5p/VASP regulation	[184]
BDNF-AS	ENSG00000245573	RNH1/TRIM21/mTOR pathway	[172]
BLACAT1	ENSG00000281406	miR-503/Bcl-2 regulation	[185]
CCAT2	ENSG00000280997	Hyperactivation of ERK/MAPK pathway	[162]
CYTOR	ENSG00000222041	miR-125a-5p/SRF regulation	[127]
DILA1	ENST00000435697.1	cyclin D1 binding/pRb phosphorylation	[174]
DSCAM-AS1	ENSG00000235123	Interaction with hnRNPL	[164]
		miR-137/EPS8 regulation	[165]
FAM83H-AS1	ENSG00000203499	--	[186]
H19	ENSG00000130600	Wnt/β-catenin pathway and EMT process	[150]
		Induction of autophagy	[85]
		Notch and c-Met signaling, *ESR1* regulation	[149]
HNF1-AS1	ENSG00000241388	miR-363/SERTAD3 regulation/TGF-ß/Smad pathway regulation	[139]
HOTAIR	ENSG00000228630	Promotion of ligand-independent ER activation	[166]
HOTAIRM1	ENSG00000233429	Upregulation of HOXA1	[187]
LINC-ROR	ENSG00000258609	Inhibition of autophagy	[148]
		MAPK/ERK pathway	[147]
		Epithelial-mesenchymal transition	[33]
LINP1	ENSG00000223784	Reduces *ESR1* expression	[171]
LOL	ENST00000456526	let-7 inhibition	[188]
MAFG-AS1	ENSG00000265688	miR-339-5p/CDK2 regulation	[173]
TMPO-AS1	ENSG00000257167	stabilizes *ESR1* mRNA/*ESR1* regulation	[189]
UCA1	ENSG00000214049	mTOR pathway	[156]
		Feedback loop with miR-18a/HIF1A	[157]
		Wnt/β-catenin pathway	[155]
		PI3K/AKT pathway	[159]
		--	[157]
Downregulated			
ADAMTS9-AS2	ENSG00000241684	miR-130a-5p/PTEN regulation	[178]
GAS5	ENSG00000234741	miR-222/PTEN regulation	[179]
LINC00472	ENSG00000233237	ERα-inducible/NF-ΚB regulation	[190]
LINC00894-002	ENST00000444489	TGF-β2 pathway	[191]

Note: Regultion (up or down) of lncRNAs in tamoxifen resistant breast cancer cells is indicated as reported. (--) information not provided in the study.

## 7. Final Considerations

**Figure 4:**
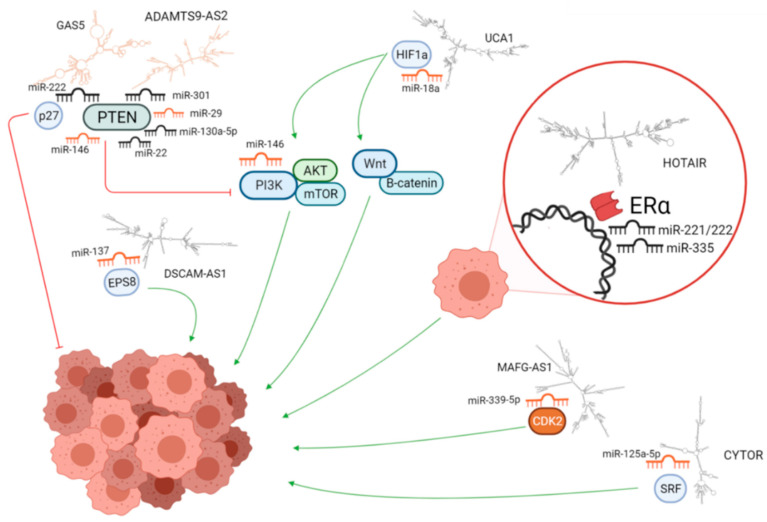
miRNAs, lncRNAs, and target mRNA differentially expressed in resistant breast cancer cells lines. Upregulated miRNAs and lncRNAs are in black, while those downregulated are in orange. Structures of lncRNAs were predicted using the RNAfold tool in the ViennaRNA Package 2.0 and drawn using MFE plain structure drawing. The figure does not contain all regulatory molecules found in this study. The molecules are not to scale.

In this literature review, we identified 75 microRNAs and 22 lncRNAs associated with Tam resistance. The mechanisms of Tam resistance are complex and include multiple actors involved in different functions and signaling pathways. The ncRNAs participate in important regulatory processes and evidence thus far indicates that they can contribute to Tam resistance (Figure 4). 

These ncRNAs can have multiple targets that are able to participate in more than one regulatory pathway. Their action may be modulated by the relative expression levels of their different targets in the cancer cell. In addition, microRNAs and lncRNAs may crosstalk by targeting overlapping sets of genes and jointly contributing to the regulation of the signaling pathways involved in tamoxifen resistance. Furthermore, lncRNAs can act as sponges for miRNAs, dampening their action on target transcripts. These interactions are thus complex and the better we identify potential regulators and clarify their mechanisms of action, the better we will understand their impact on the resistance process. 

Use of genomic testing in early-stage hormone receptor-positive breast cancer to aid the therapeutic choice has become a reality in the clinic, but ncRNAs are underexploited in this respect. The increasing volume of information about the pertinence of ncRNAs, mainly microRNAs, suggests that these molecules should be further studied for inclusion in future panels. These panels can, in addition to contributing to a more incisive diagnosis, guide the best treatment through predictions of the therapeutic response for each patient. The stability of miRNAs in blood [192] may also facilitate detection of emergent resistant tumor subclones during treatment.

As for the use of ncRNAs in therapeutic strategies, direct applications to patient care are even further away. However, initial successes of RNA interference strategies are promising in this respect, including miRNA mimics and inhibitors (antimiRs) [193]. From the great number of studies identifying miRNAs as potential therapeutic targets, some have led to clinical trials. For example, a mimic of miR-34 is in phase I clinical trials for cancer treatment and antimiRs for miR-122 have reached phase II trials for treating hepatitis [194,195]. This information gives hope for the clinical usefulness of ncRNA modulators that may interfere in tamoxifen resistance.

In conclusion, knowledge about the roles of ncRNAs in Tam resistance may help to elucidate this serious problem that influences the treatment and prognosis of thousands of patients. Additionally, this knowledge can be used to improve biomarker panels for molecular diagnosis and treatment prediction, in order to individualize the therapeutic approach and give the patient more accurate treatment, for example, identifying patients at greater risk of relapse due to the expression patterns of non-coding RNAs that impact the response to Tam. Additionally, this knowledge may, in the future, be used to improve treatment options and possibly also to design therapeutic strategies to bypass mechanisms of resistance.
